# Whi3, an *S. cerevisiae* RNA-Binding Protein, Is a Component of Stress Granules That Regulates Levels of Its Target mRNAs

**DOI:** 10.1371/journal.pone.0084060

**Published:** 2013-12-27

**Authors:** Kristen J. Holmes, Daniel M. Klass, Evan L. Guiney, Martha S. Cyert

**Affiliations:** 1 Department of Biology, Stanford University, Stanford, California, United States of America; 2 Department of Biochemistry, Stanford University School of Medicine, Stanford, California, United States of America; 3 Howard Hughes Medical Institute, Stanford University School of Medicine, Stanford, California, United States of America; Dartmouth College, United States of America

## Abstract

RNA binding proteins (RBPs) are vital to the regulation of mRNA transcripts, and can alter mRNA localization, degradation, translation, and storage. Whi3 was originally identified in a screen for small cell size mutants, and has since been characterized as an RBP. The identification of Whi3-interacting mRNAs involved in mediating cellular responses to stress suggested that Whi3 might be involved in stress-responsive RNA processing. We show that Whi3 localizes to stress granules in response to glucose deprivation or heat shock. The kinetics and pattern of Whi3 localization in response to a range of temperatures were subtly but distinctly different from those of known components of RNA processing granules. Deletion of Whi3 resulted in an increase in the relative abundance of Whi3 target RNAs, either in the presence or absence of heat shock. Increased levels of the *CLN3* mRNA in *whi3*Δ cells may explain their decreased cell size. Another mRNA target of Whi3 encodes the zinc-responsive transcription factor Zap1, suggesting a role for Whi3 in response to zinc stress. Indeed, we found that *whi3*Δ cells have enhanced sensitivity to zinc toxicity. Together our results suggest an expanded model for Whi3 function: in addition to its role as a regulator of the cell cycle, Whi3 may have a role in stress-dependent RNA processing and responses to a variety of stress conditions.

## Introduction

The budding yeast *Saccharomyces cerevisiae* adapts to cellular stress by arresting the cell cycle and altering a variety of RNA metabolic processes. Stalled translational initiation causes increased transcript deadenylation, decapping and decay, and promotes the formation of highly conserved RNA degradation structures called processing bodies (P-bodies) [1], [2]. In yeast, P-bodies are hubs of RNA degradation during both steady-state and stress conditions. P-body-associated proteins are primarily enhancers of RNA decapping and decay [2]–[4]. During stress, P-body formation is enhanced and P-bodies can nucleate the formation of related structures called stress granules. Though P-bodies and stress granules share overlapping functions and protein components, stress granules only appear during periods of cellular stress and contain a wider array of RNA processing proteins than P-bodies, such as translation initiation factors, 40 S ribosomal subunits, and stability-promoting RNA binding proteins [5]–[7]. This broad repertoire of RNA processing proteins allows stress granules to tailor stress responses by promoting storage or translation of particular mRNAs, while other mRNAs undergo decay [3], [6].

Whi3 is an RNA binding protein that contains a conserved RNA recognition motif (RRM) at its C-terminal end and was originally identified in a screen for small cell size mutants [8], [9]. Other functional regions of Whi3 include a Cdc28 recognition motif required for interaction with the cyclin-dependent kinase Cdc28, and a glutamine-rich (Q-rich) region that has been minimally characterized [8]–[10]. Microarray experiments have identified approximately 300 Whi3 target mRNAs, including transcripts involved in cell cycle regulation, cell wall biogenesis, and transcription factors [11], [12]. Whi3 target mRNAs are enriched for tetranucleotide (U)GCAU motifs, which are required for interaction with Whi3 [11], [13].

The best-characterized Whi3-interacting mRNA is the yeast cell cycle regulator *CLN3* [11], [14]. Cln3 binds to its Cdk partner Cdc28 to promote cell cycle entry, and several *whi3*Δ phenotypes depend upon the presence of Cln3, including small size, meiotic entry, pseudohyphal growth, and invasive growth [8], [10], [14], [15]. Mutation of the (U)GCAU motifs in *CLN3* mRNA, preventing Whi3 binding, results in a small-cell phenotype equivalent to that of *whi3*Δ cells, indicating that Whi3-mediated cell size regulation is dependent upon the interaction between Whi3 and *CLN3* [11]. A current model of Whi3 function in *S. cerevisiae* suggests that Whi3 sequesters *CLN3* mRNA and Cdc28 protein in the cytoplasm to prevent premature cell cycle entry [8], [10]–[12], [14], [16].

Though the well-characterized interaction with *CLN3* transcripts suggests that the biological function of Whi3 involves RNA regulation, most Whi3 target mRNAs are not cell cycle-regulated, suggesting that Whi3 may have functions unrelated to the cell cycle. For example, Whi3 binds to a variety of mRNAs required for cell wall maintenance, and *whi3*Δ cells are sensitive to cell wall stressors such as Congo red and calcofluor white; these sensitivity phenotypes are largely dependent upon Whi3-mRNA interactions [11]. Whi3 interacts with mRNA targets implicated in many other biological processes, and the physiological significance of most of these Whi3-mRNA interactions has not yet been investigated.

The identification of Whi3 mRNA targets involved in cell cycle regulation and RNA processing, both of which change in response to stress, suggested the possibility that Whi3 might be involved in stress-responsive RNA processing. Whi3 shares a number of characteristics with known stress granule-associated proteins. The Whi3 RRM shares homology with RNA binding domains of known stress granule components Pub1 and Ngr1 [17]. Whi3 has been reported to form cytosolic granules [10]. Proteomic data suggest that Whi3 protein molecules physically interact with one another, with the stress granule component Pub1, and with several RNA processing factors, including Cdc39, Ded1, and Def1 [18]. The Whi3 Q-rich region shares homology with the Q-rich region of P-body protein Lsm4, and weakly aligns with that of Edc3 [6], [17]. These facts suggest that Whi3 might be a novel component of stress granules, a hypothesis that was tested in this study.

## Results

### Whi3 Localizes to Stress Granules during Glucose Deprivation

**Figure 1:**
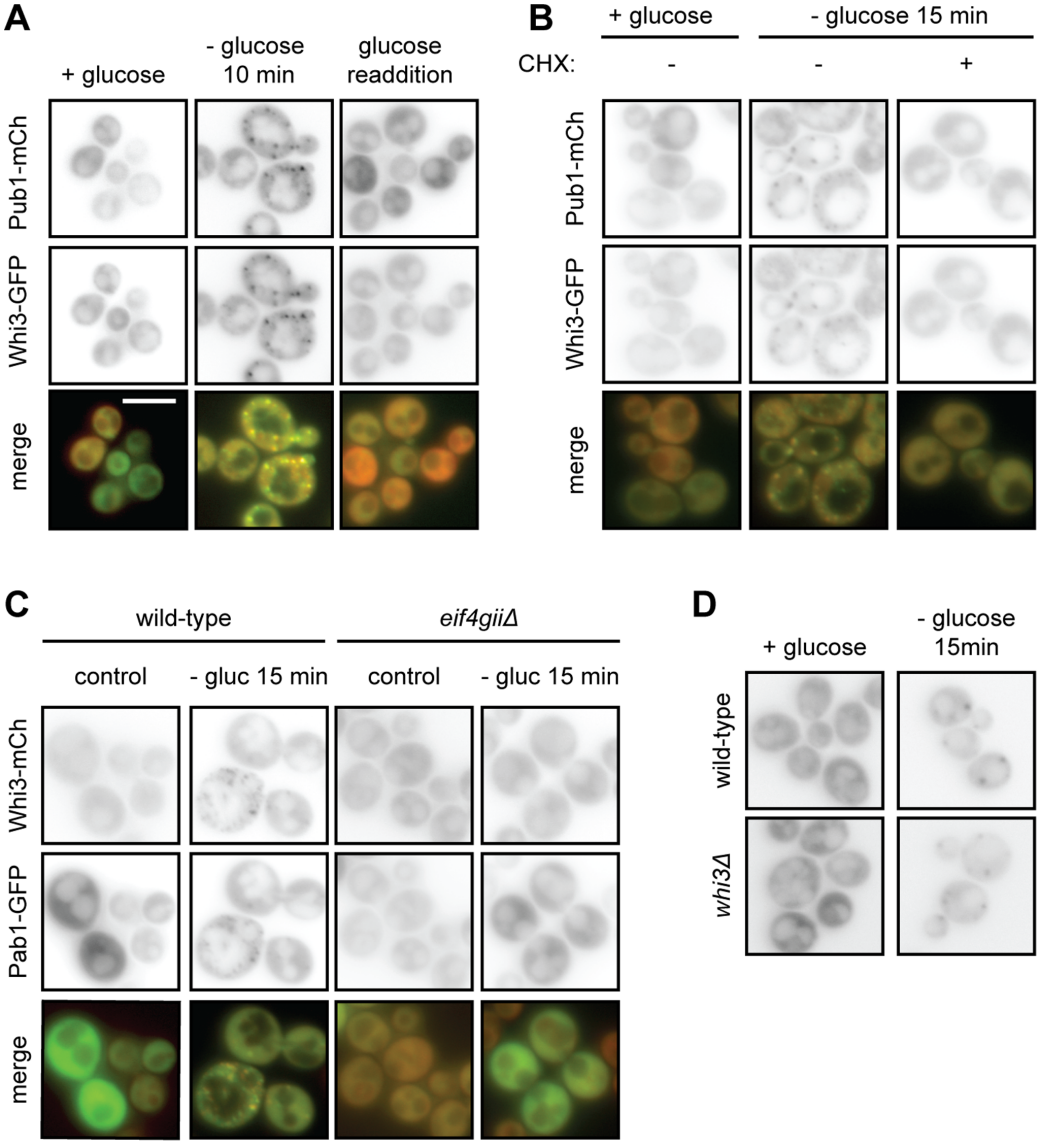
Whi3 localizes to stress granules during glucose deprivation. (A) Cells coexpressing Whi3-GFP and stress granule marker Pub1-mCherry were deprived of glucose for 10 minutes. Glucose deprivation caused Whi3 and Pub1 to colocalize in distinct cytoplasmic foci. Readdition of glucose caused foci to dissolve. Scale bar represents 5 µm. (B) Treatment with cycloheximide (CHX) inhibits stress granule formation by preventing polysome disassembly. Unlike cells treated with drug vehicle alone, cells treated with CHX form neither Pub1-mCh nor Whi3-GFP foci. (C) Cells lacking the scaffolding protein eIF4GII do not form stress granules. Neither Whi3-mCh nor Pab1-GFP forms foci in *eif4gii*Δ cells. (D) Stress granule marker Pub1-mCh is still able to localize to foci in the absence of Whi3.

To investigate the molecular function of Whi3, we examined its localization using a fully functional *WHI3-GFP* fusion gene, integrated at the *WHI3* locus to maintain endogenous regulation of its expression. In cells grown under standard conditions at 30°C Whi3-GFP exhibited diffuse cytosolic localization (Figure 1A), similar to previous observations in fixed cells [14], [19]. Because Whi3 shares characteristics with proteins that localize to stress granules, and is reported to interact with Pub1, a known component of stress granules [18], we also examined Whi3-GFP localization in cells deprived of glucose, a condition that induces stress-granule formation in yeast [5], [20]–[24]. Under these conditions, Whi3 localized to distinct cytoplasmic foci, colocalizing with Pub1-mCherry (Figure 1A). Upon the re-addition of glucose, both Whi3-GFP and Pub1-mCherry returned to a diffuse localization pattern (Figure 1A). This reversible, stress-dependent, punctate localization suggests that, like Pub1-mCherry, Whi3 is a component of stress granules.

To further test this hypothesis, we determined Whi3 localization under conditions that inhibit stress granule formation. First, cells were incubated with cycloheximide, which inhibits stress granule formation by preventing polysome disassembly [5], [25], [26]. In cycloheximide-treated cells that were deprived of glucose, Pab1-GFP and Whi3-mCherry both exhibited diffuse cytosolic localization and failed to form distinct foci (Figure 1B). Similarly, neither Pab1-GFP nor Whi3-mCherry localized to foci after glucose deprivation in *eif4gii*Δ cells, which are unable to form stress granules under these conditions (Figure 1C) [5]. Taken together, these data indicate that Whi3 is a previously unrecognized component of stress granules in glucose-deprived cells.

### Whi3 is not Required for Stress Granule Formation

To test whether Whi3, like eIF4GII [5], is required for stress granule formation, we examined Pub1-mCherry localization wild-type and *whi3*Δ cells after glucose starvation. Pub1-mCherry localized to foci in both strains, indicating that Whi3 is not a required structural component of stress granules that form during nutrient deprivation (Figure 1D).

### Whi3 Localizes to Stress Granules during Heat Shock

**Figure 2:**
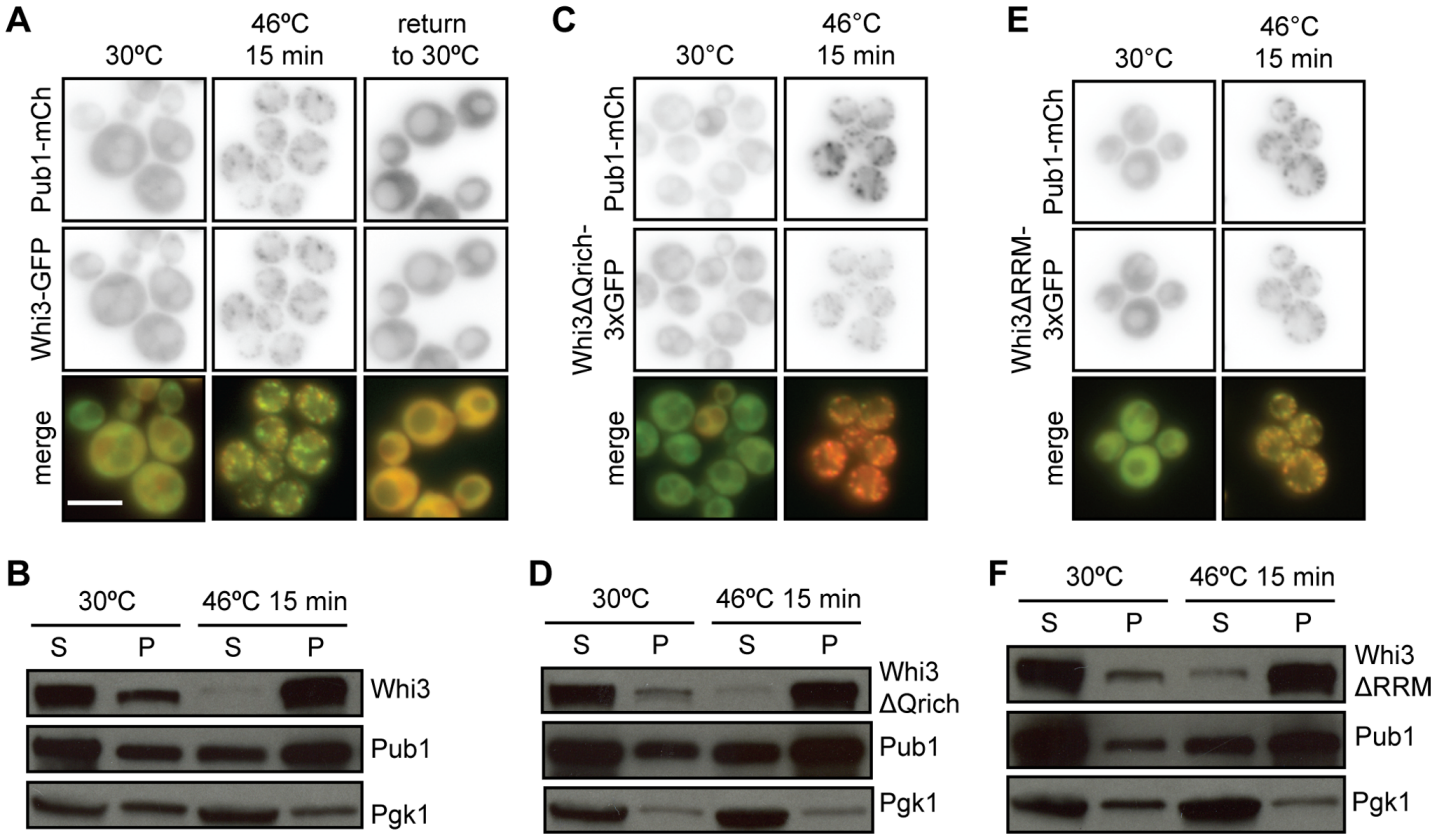
Whi3 localizes to stress granules during heat shock. (A) Cells co-expressing Pub1-mCh and Whi3-GFP were heat shocked at 46°C for 15 minutes. Heat shock caused Whi3 and Pub1 to colocalize in distinct cytoplasmic foci. When cells were returned to 30°C, these foci dissolved. Scale bar represents 5 µm. (B) Pelleting assay results support the observation that Whi3 is enriched in insoluble stress granules during heat shock. Both Whi3FL-3xGFP and Pub1-mCh are enriched in soluble (S) fractions at 30°C, but become enriched in the insoluble pellet (P) fraction during heat shock at 46°C. Housekeeping gene Pgk1 does not change solubility in response to stress. (C) Whi3ΔQrich-3xGFP colocalizes with stress granule marker Pub1-mCherry during heat shock. (D) Whi3ΔQrich exhibits a similar solubility profile to Whi3FL-3xGFP. (E) Whi3ΔRRM-3xGFP colocalizes with stress granule marker Pub1-mCherry during heat shock. (F) Whi3ΔRRM exhibits a similar solubility profile to Whi3FL-3xGFP.

In yeast, stress granules form in response to heat shock as well as glucose deprivation, although the protein composition of stress granules varies under these two conditions [3], [21], [26], [27]. In cells exposed to 15 minutes of heat shock at 46°C (Figure 2A), Whi3-GFP displayed punctate distribution and colocalization with Pub1-mCherry-containing foci, which was reversed after cells were returned to 30°C. Whi3-mCherry also colocalized in foci with Pab1-GFP, a previously characterized component of stress granules, under these conditions (data not shown). These observations suggest that Whi3 localizes to stress granules in response to multiple stress conditions.

### The RRM and Glutamine-rich Domains of Whi3 are not Required for its Localization to Stress Granules During Heat Shock

Many protein components of stress granules contain an RNA-binding domain and/or a glutamine-rich domain, which mediates stress granule aggregation [6], [28]. To determine if the glutamine-rich region of Whi3, which has no known function [6], [9], [28], is required for Whi3 localization to stress granules, we compared the localization of full-length, GFP-tagged Whi3 (Whi3-3xGFP) with that of a Whi3 mutant lacking the glutamine-rich region (Whi3ΔQrich-3xGFP). Whi3ΔQrich colocalized with Pub1-mCherry in stress granules in response to heat shock (Figure 2C) and glucose deprivation (data not shown), indicating that the glutamine-rich region is not required for Whi3 localization to stress granules.

Whi3 also contains an RNA-binding domain termed an RNA recognition motif (RRM), and binds to a specific set of cellular mRNAs, including *CLN3* [11], [14]. To determine whether RNA binding is required for Whi3 localization to stress granules, we constructed a strain with *WHI3*Δ*RRM-3xGFP*, lacking the RRM motif, integrated into the genome. Whi3ΔRRM-3xGFP localized to foci in heat-shocked cells, indicating that RNA binding is not required for Whi3 localization to stress granules under these conditions (Figure 2E).

### Differential Centrifugation Confirms Stress-dependent Change in Whi3 Localization

Because P-bodies and stress granules are composed of large RNP aggregates, they pellet at 10,000×g force in cytosolic extracts [4], [26]. To extend our microscopic observations, we examined the sedimentation characteristics of Whi3-3xGFP, Whi3ΔQrich-3xGFP, and Whi3ΔRRM-3xGFP in cell extracts. In extracts of cells grown at 30°C and centrifuged at 10,000×g, the majority of Whi3-3xGFP remained in the cytoplasmic supernatant fraction, and a small amount appeared in the insoluble pellet fraction. In contrast, in extracts of cells exposed to 46°C for 15 minutes, nearly all of the Whi3-3xGFP distributed in the pellet fraction (Figure 2B). The behavior of Whi3ΔQrich-3xGFP and Whi3ΔRRM-3xGFP in this assay was indistinguishable from that of Whi3-3xGFP (Figure 2D, 2F) and similar to that of a known component of stress granules, Pub1-mCherry (Figure 2B, 2E, 2F). In contrast, the sedimentation of phosphoglycerate kinase, Pgk1, a cytosolic metabolic enzyme that does not localize to stress granules, did not change in extracts of stressed cells (Figure 2B, 2E, 2F). These results support the conclusion that Whi3-3xGFP, Whi3ΔQrich-3xGFP, and Whi3ΔRRM-3xGFP specifically localize to stress granules in cells exposed heat shock at 46°C.

### Whi3 Colocalizes with P-bodies during Mild Heat Shock

**Figure 3:**
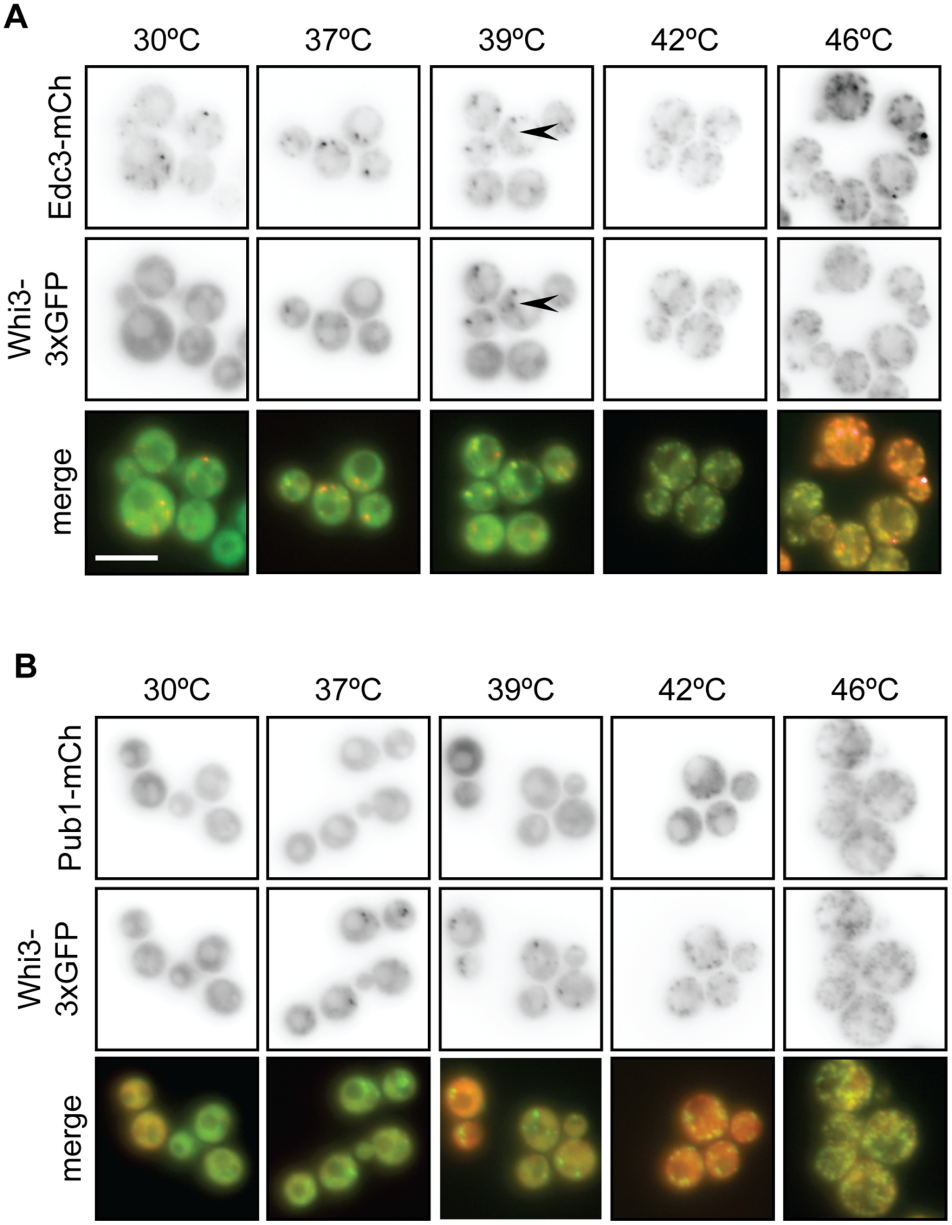
Whi3 nucleates stress granules during mild heat shock. (A) Cells coexpressing Whi3FL-3xGFP and P-body marker Edc3-mCh were incubated at various temperatures for 15 minutes. Whi3 does not colocalize with P-bodies during non-stress conditions (30°C). As temperatures increase, both Whi3 and Edc3 form foci. Though some Whi3 foci are distinct from Edc3 foci (arrowheads), colocalization between Whi3 and Edc3 increases as temperature increases. Scale bar represents 5 µm. (B) The experiment described in (A) was repeated in cells coexpressing Whi3FL-3xGFP and stress granule marker Pub1-mCh. Though Whi3 begins localizing to foci at 37°C, Pub1 foci do not form until 42°C. Pub1 and Whi3 foci colocalize at temperatures at or above 42°C.

**Figure 4:**
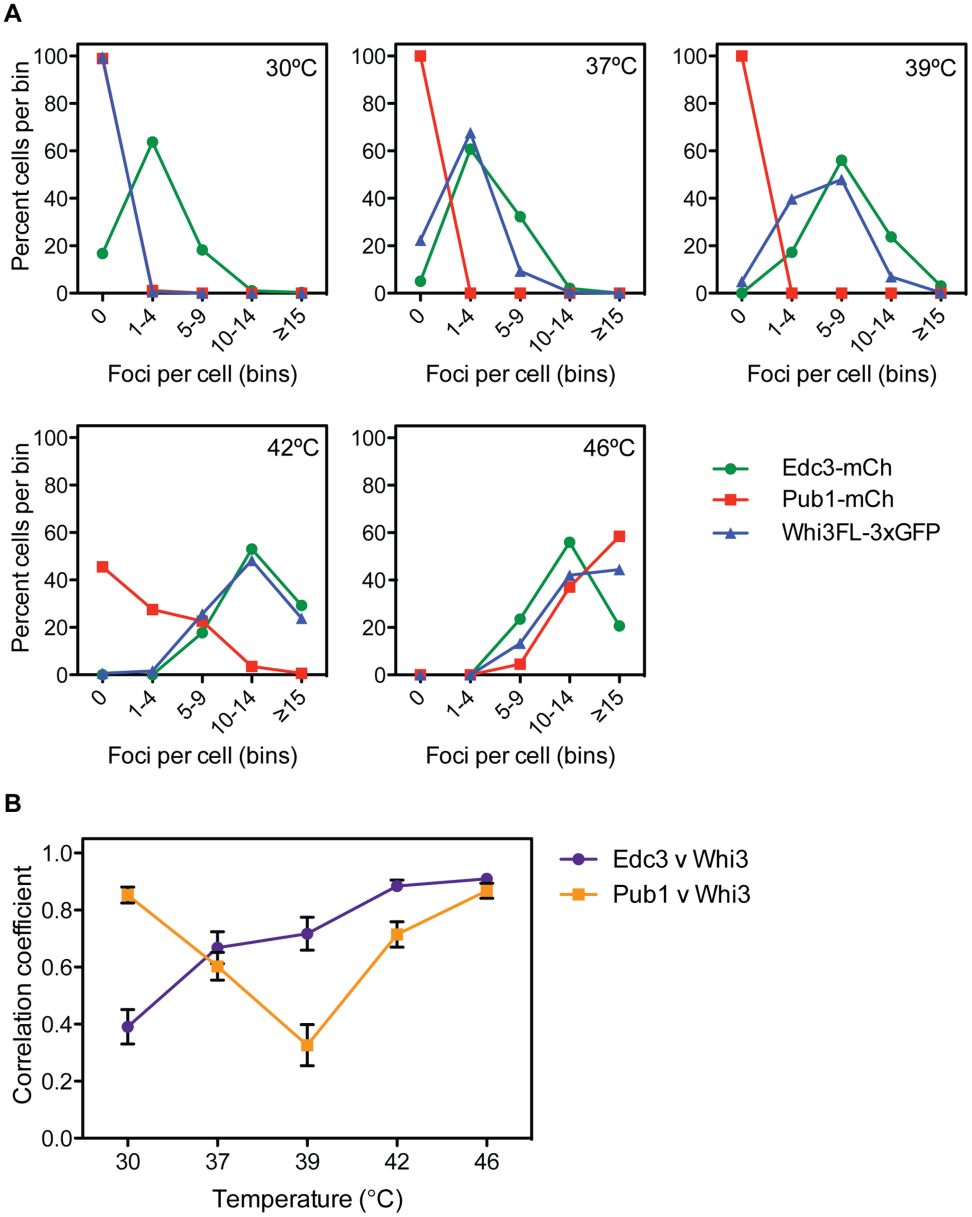
Quantification of temperature course data. (A) Double-blinded quantification of data from experiments presented in Figure 3. Whi3-3xGFP, Pub1-mCh, and Edc3-mCh foci were manually counted using a binning method, as automated analysis was not possible. Edc3-mCh forms foci at all temperatures, though foci become enriched as temperatures increase. Pub1-mCh remains diffusely cytosolic until 42°C, at which point it localizes to foci. Whi3FL-3xGFP forms increasing numbers of foci starting at 37°C. An average of 300 cells were analyzed for each protein at each temperature. (B) Double-blinded colocalization analysis of experiments presented in Figure 3. Using ImageJ software [49], a line was drawn through a cell, avoiding the vacuole, and a plot of signal intensity was generated. The analysis was repeated in the corresponding image and a Pearson’s correlation coefficient between the two lines was calculated. Data presented are the average of 20 cells; error bars represent SEM. Pub1-mCh and Whi3FL-3xGFP exhibit high correlation at 30°C when both are diffusely cytosolic, but correlation decreases at 37°C and 39°C when Whi3 begins localizing to foci. Pub1 and Whi3 correlate more robustly once Pub1 foci begin to form at 42°C. Edc3-mCh and Whi3FL-3xGFP correlate poorly at 30°C when Edc3 is in foci, but become more highly correlated as temperatures increase.

To determine whether Whi3 is a component of P-bodies, we compared the localization of Whi3-3xGFP and Edc3-mCherry, a component of P-bodies, in cells exposed to a range of temperatures. At 30°C, when P-bodies, but not stress granules, are present, most cells contained several (between one and four) Edc3-mCherry-containing puncta (Figures 3A and 4A). In contrast, Whi3-3xGFP displayed diffuse cytosolic staining at 30°C and did not localize to Edc3-mCherry-containing foci (Figure 4B), indicating that Whi3 is not a component of P-bodies in unstressed cells.

In cells exposed to mild heat shock (37°C) the number of Edc3-mCherry-containing P-bodies increased (Figures 3A and 4A), and Whi3-3xGFP-containing foci were observed, with most cells exhibiting a maximum of four such puncta (Figures 3A, 3B, 4A). Some foci contained both Edc3-mCherry and Whi3-3xGFP at 37°C, and colocalization of these proteins increased compared to 30°C (Figure 4B), suggesting that Whi3 is recruited to stress-enhanced P-bodies during mild stress. This trend continued at 39°C; Whi3-3xGFP and Edc3-mCherry foci became more abundant (Figures 3A, 4A), and colocalization of these proteins increased (Figure 4B). Also, at 39°C, it was apparent that some Whi3-3xGFP containing puncta were distinct from Edc3-mCherry foci (Figure 3A, arrowhead). The number of Whi3-3xGFP and Edc3-mCherry-containing foci continued to increase at 42°C and 46°C, and their colocalization was similarly enhanced at these temperatures (Figures 3A, 4A, 4B). Thus, in response to increasingly severe heat shock, stress-enhanced P-bodies became more abundant. In parallel, the distribution of Whi3 became more punctate, and its association with stress-enhanced P-bodies increased.

We similarly examined the distribution of Pub1-mCherry, a component of stress granules, and Whi3-3xGFP over a range of temperatures. Pub1-mCherry remained diffusely cytosolic at 30, 37 and 39°C (Figure 3B). At 39°C, colocalization of Pub1-mCherry and Whi3-3xGFP was low, as 95% of cells contained one or more Whi3-3xGFP-containing puncta, while Pub1-mCherry remained diffusely distributed (Figure 3B, Figure 4A, 4B). However, at 42°C, Pub1-mCherry-containing stress granules were observed, and became more numerous in cells at 46°C (Figure 3B, 4A); colocalization of Whi3-3xGFP and Pub1-mCherry increased at these temperatures (Figure 4B). During the most severe heat shock (46°C), both Edc3-mCherry and Pub1-mCherry colocalized with Whi3-3xGFP, consistent with previous findings that components of P-bodies and stress granules colocalize during stress (Figure 3, Figure 4, and data not shown) [5], [26]. Taken together, these data show that Whi3 localizes to RNP aggregates during both mild and severe heat shock, and is thus a component of stress-enhanced P-bodies and/or stress granules. Furthermore, Whi3 may define a new class of RNP aggregates that does not contain Edc3 or Pub1.

### Comparison of Data from Two Independent Isolations of Whi3-bound mRNAs Reveals a High-confidence Whi3 Target Set

**Figure 5:**
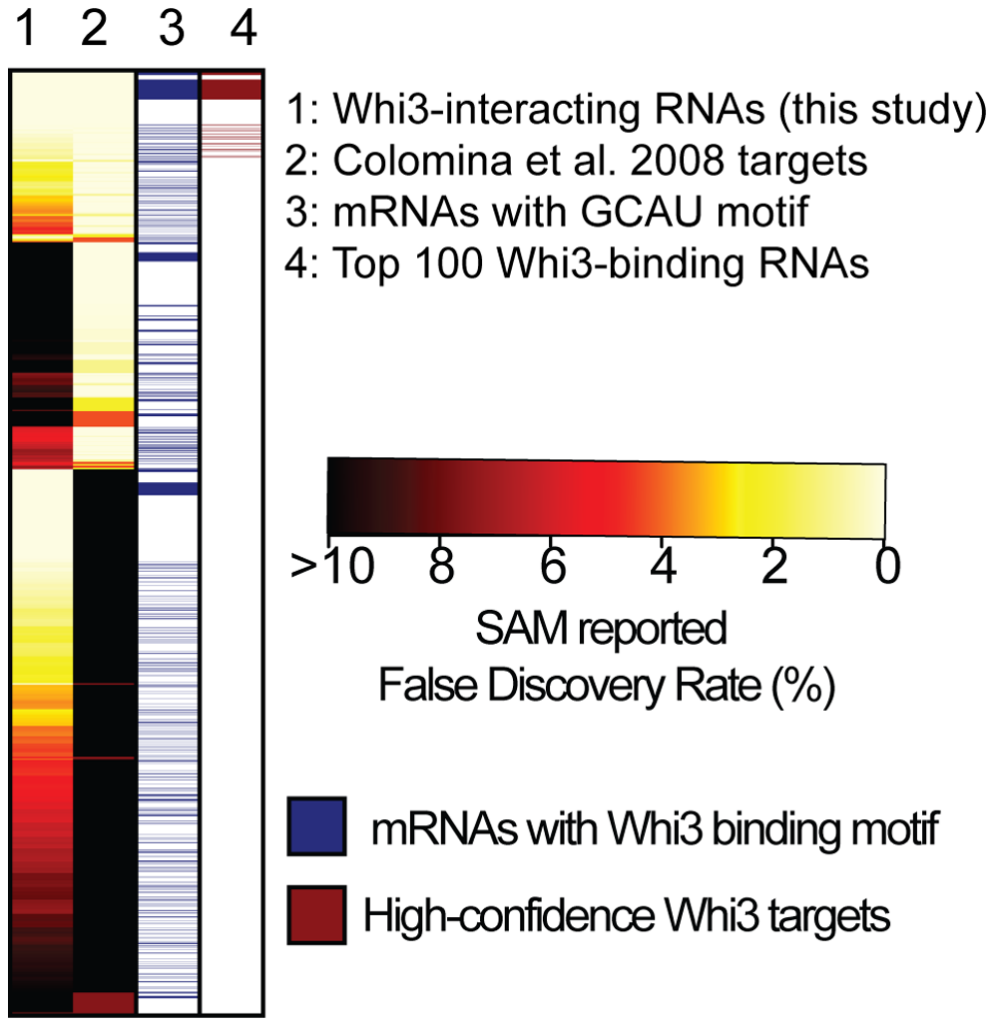
Comparison of multiple data sets reveals a high-confidence Whi3 RNA target set. Heat map showing the overlap among Whi3 targets identified in the Whi3 IP presented in this study or from the Colomina data set [11]. Each row is a Whi3 target mRNA identified in at least one of the two data sets with a false discovery rate (FDR) of <10%. The first two columns show the SAM reported false discovery rates for each mRNA in either our Whi3 IP data set or the Colomina data set, with FDRs ranging from >10% in black through red, orange, yellow and 0% in light yellow. The third column indicates in blue which mRNAs contain the (U)GCAU Whi3 interaction motif. The fourth column indicates in red which mRNAs we defined as the top 100 high-confidence Whi3-interacting RNAs.

Whi3 regulates cell size by binding to the mRNA that encodes Cln3, a G_1_ cyclin, and specifically recognizes mRNAs that contain (U)GCAU repeats [11], [13]. To identify additional Whi3 mRNA targets, we purified Whi3 from cell extracts as previously described [29], but used a one-class Significance Analysis of Microarrays (SAM) algorithm to identify targets, which is similar to a t-test [29], [30]. This analysis revealed 418 Whi3 mRNA targets with a false discovery rate of <1% (Figure 5 and Table S1). These targets were then compared to those from a previously published set of Whi3-binding mRNAs which, when reanalyzed using SAM analysis identified 1,018 Whi3 target mRNAs with a false discovery rate of <1% (Figure 5 and Table S1) [11]. 269 mRNAs were common to both data sets, of which 100 also contained one or more copies of the Whi3-binding motif, (U)GCAU [11], [13]. These 100 mRNAs constitute a high-confidence set of Whi3 target mRNAs (Figure 5 and Table S1).

### Enrichment of Gene Ontology Terms among Whi3 mRNA Target Sets

To gain insight into the function of Whi3 in the post-transcriptional regulatory network, we analyzed the enrichment of Gene Ontology (GO) terms among the Whi3 mRNA targets (Figure S1). GO term enrichment was calculated using the GOstats package in R, the statistical analysis software (http://www.r-project.org), and the resulting p-values were corrected for multiple hypothesis testing with the Bonferroni correction [31]. The GO term analysis revealed that Whi3 binds to mRNAs encoding proteins involved in regulating transcription, the cell cycle, and the response to environmental stimuli. Specifically, Whi3 bound to mRNAs encoding numerous transcription factors (e.g., *NRG1*, a regulator of glucose repression), several cyclins (e.g., *CLN2*, *CLN3*), proteins involved in the response to pheromone arrest (e.g., *POG1*), in regulation of cell size and ribosomal RNA transcription, and in zinc ion transport, including the zinc-responsive transcription factor Zap1.

### Loss of Whi3 does not Significantly Affect the Translation of Target mRNAs

**Figure 6:**
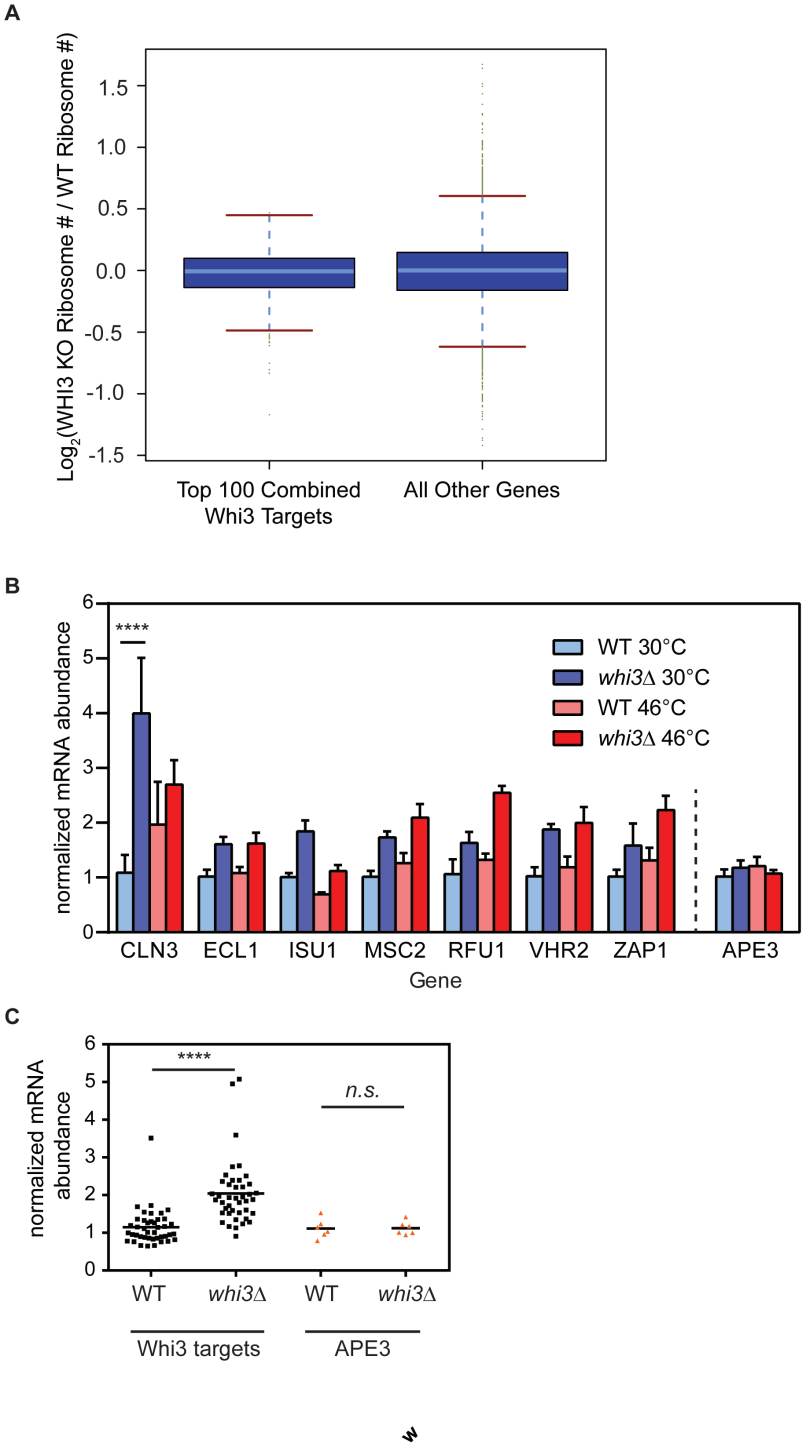
Whi3 changes steady-state levels of its target mRNAs without affecting translation rates. (A) Boxplot of log base 2 of the fold change in the average number of ribosomes bound to each mRNA in a Whi3 deletion versus a wild-type strain. The top 100 high-confidence Whi3 targets are plotted on the left, compared to all other mRNAs on the right, showing that there is no significant difference in the change in ribosome number in response to Whi3 deletion between these two sets of mRNAs. (B) Wild-type or *whi3*Δ cells were grown to mid-log phase in YPD, then incubated for 15 minutes at 30°C or 46°C. RT-PCR analysis examined levels of several RNA targets identified in Figure 5. Data are averages of three biological replicates; error bars represent SEM. ****, p<0.0001, Sidak’s post test correction for multiple comparison. (C) Pooled data from B. Normalized mRNA abundance is plotted for Whi3 targets or *APE3*, grouped by strain background, wild-type or *whi3*Δ. ****, p<0.0001, 2-way ANOVA.

To determine whether Whi3 regulates translation of its target RNAs, we performed genome-wide translation analysis as described previously [32]. Briefly, we compared the average number of ribosomes bound to a given mRNA species (i.e. ribosome number) between a wild-type strain and a *whi3*Δ strain grown to mid-log phase in YPD. We found no significant difference in the Whi3-dependent change in ribosome number for the top 100 high-confidence Whi3 targets compared to all other genes (Figure 6A), suggesting that Whi3 does not regulate translation of the mRNAs to which it is bound.

### Whi3 Regulates the Abundance of its Target mRNAs

To examine possible effects of Whi3 on the abundance of its mRNA targets, quantitative RT-PCR analysis was performed on steady-state mRNA samples from wild-type and *whi3*Δ cells at both 30°C and 46°C. mRNAs examined included *CLN3*, the best-characterized Whi3-binding mRNA, six additional mRNAs from the high-confidence set of Whi3 targets discussed above (*ECL1, ISU1, MSC2, RFU1, VHR2, ZAP1*), and, as a negative control, *APE3* mRNA, which contains no (U)GCAU motifs and is not bound by Whi3. Within each sample, individual mRNAs were normalized to the level of actin mRNA to determine abundance; there was no significant difference between actin levels at 30°C or 46°C (data not shown). Then, the amount of each mRNA in *whi3*Δ cells relative to its level in wild-type cells grown at 30°C was determined (Figure 6B).

At both 30°C and 46°C, all Whi3 target mRNAs, but not *APE3*, displayed greater abundance in *whi3*Δ than in wild-type cells (Figure 6B), and this effect was highly significant (Figure 6C, p<0.001, 2-way ANOVA). At 30°C, the extent to which each mRNA increased in *whi3*Δ cells relative to wild-type varied from 1.5-fold (*ECL1*, *RFU1*, *ZAP1*) to as much as 3.5-fold (*CLN3*). The effect of temperature stress on mRNA targets of Whi3 was less consistent. Though the abundance of each of the tested RNA targets of Whi3 was elevated in *whi3*Δ relative to wild-type cells, some targets decreased in overall abundance at 46°C relative to 30°C (*ISU1*), while others remained stable or increased (*CLN1*, *ECL1*, *MSC2*, *VHR2*) (Figure 6B). Taken together, these results suggest that Whi3 decreases steady state levels of its target mRNAs under both normal and stress conditions. However, the degree to which Whi3 affects each target mRNA varies, as does the response of each target mRNA to stress.

*CLN3* mRNA abundance increased approximately 3.5-fold in *whi3*Δ cells compared to wild-type cells at 30°C (p<0.0001, 2-way ANOVA with multiple comparisons, Figure 6B), and was the largest change observed in our sample. Increased levels of *CLN3* mRNA could explain why *whi3*Δ cells proceed more rapidly through the cell cycle than wild-type cells, leading to a small cell size phenotype [8]–[11], [14]. Interestingly, at 46°C, when Whi3 is associated with stress granules, *CLN3* mRNA levels were much less affected by its absence.

### Cells Lacking Whi3 are Sensitive to Zinc

**Figure 7:**
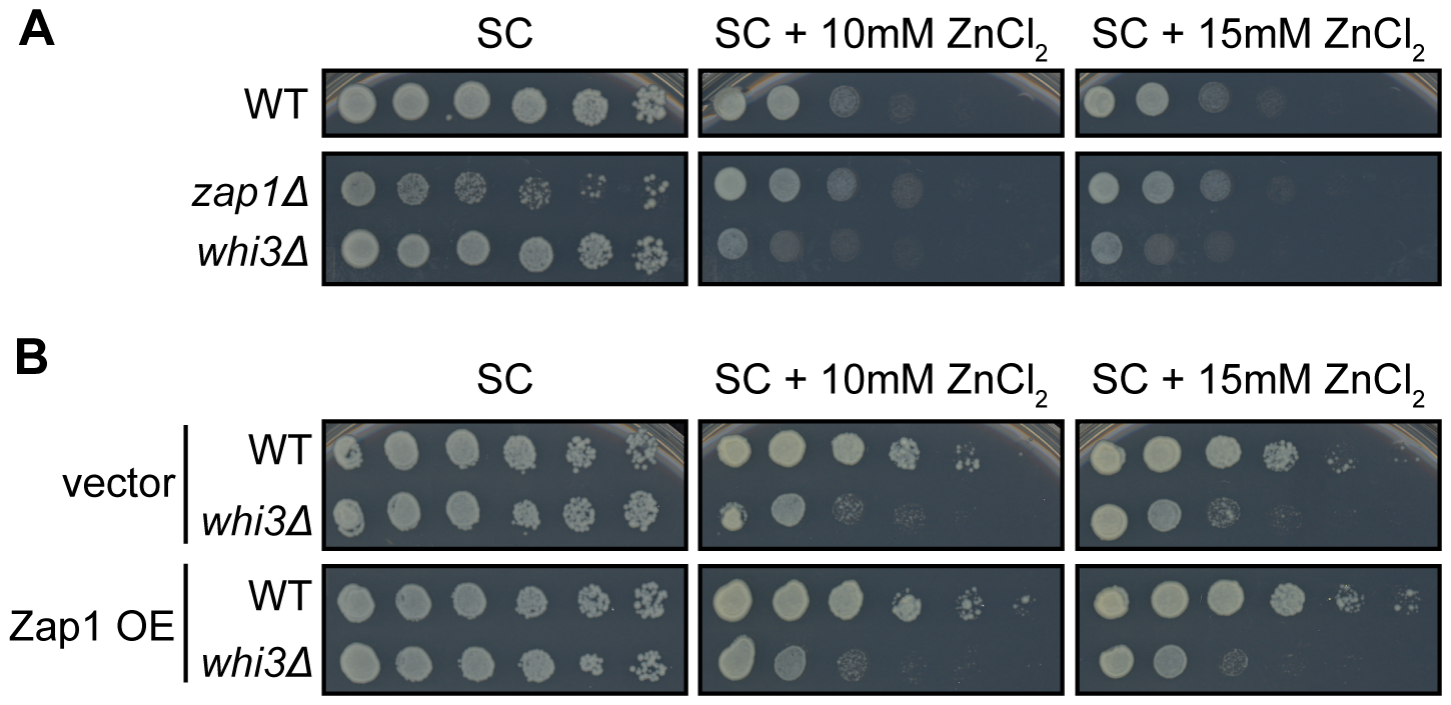
*whi3*Δ cells have a novel zinc sensitivity phenotype. (A) BY4741 cells were spotted in serial dilutions on control plates or plates containing 10 mM or 15 mM ZnCl_2_. *whi3*Δcells exhibited a novel zinc sensitivity phenotype. *zap1*Δ cells grow slowly on control media because they are zinc starved, but grow normally on plates supplemented with zinc. (B) The zinc sensitivity phenotype of *whi3*Δ cells is not exacerbated by overexpression of Zap1 (OE). *whi3*Δ cells expressing vector (V) are equally zinc-sensitive as those overexpressing Zap1.

Wild-type and *whi3*Δ cells grew equally well on control media, but growth of *whi3*Δ cells was inhibited by 10 mM or 15 mM ZnCl_2_ (Figure 7A). Zap1 is a transcriptional activator that activates genes involved in zinc metabolism in response to zinc limitation [33]–[35], and *ZAP1* mRNA is part of the high-confidence Whi3 targets described above (Figure 5). *zap1*Δ cells, which grow slowly on control media because they are zinc-starved, grow better on plates supplemented with zinc (Figure 7A).

Because Whi3 binds to several mRNAs associated with zinc-dependent phenotypes, including *MSC2* and *ZAP1,* we wondered whether the zinc sensitivity phenotype of *whi3*Δ cells could be altered by overexpression of Zap1. *whi3*Δ cells transformed with vector were sensitive to 10 mM and 15 mM zinc; this phenotype was neither enhanced nor suppressed by overexpression of Zap1 (Figure 7B). Our data cannot rule out the possibility that the zinc-sensitivity phenotype of *whi3*Δ cells is due to misregulation of multiple Whi3 mRNA targets, rather than misregulation of Zap1 alone.

## Discussion

### Whi3 is a Novel Component of Stress-enhanced P-bodies and Stress Granules

Cellular stress triggers a translational arrest, followed by polysome disassembly. The release of non-translating mRNAs into the cytoplasm triggers the formation of RNP aggregates called stress granules. Our observation that the RNA binding protein Whi3 shares several features with key stress granule components (e.g., Q-rich domain, interaction with Pub1 [9], [18]) and the observation that Whi3 localization changes from diffusely cytoplasmic to punctate in response to stress led to the hypothesis that Whi3 could be a novel component of stress granules.

We found that Whi3 localized reversibly to cytoplasmic granules in response to glucose deprivation and heat shock, and that Whi3 colocalized with known stress granule components Pub1 and Pab1 under the same conditions (Figures 1 and 2, and data not shown). Blocking stress granule assembly using cycloheximide or deletion of *eIF4GII* prevented Whi3 from localizing to stress granules during glucose deprivation, suggesting that Whi3 localization to stress-responsive foci is affected by the same factors that affect stress granule formation. Although this change in Whi3 localization depended upon stress granule formation, the reverse was not true; stress granules still formed in *whi3*Δ cells, indicating that Whi3 is not required for stress granule formation.

Neither the RRM nor glutamine-rich region of Whi3 was required for its localization to stress granules during heat shock (Figure 2). In contrast, under normal growth conditions the Q-rich region of the Whi3 homolog from the related yeast, *Ashbya gossypii,* is required for that protein to form aggregates, which allows for localization of the *CLN3* mRNA and asynchronous cell cycle regulation of nuclei within a common hyphal cytoplasm [36]. During heat shock, Whi3 may utilize both RNA dependent and Q-rich prion-type aggregation interactions to yield a robust association with stress granules that is not compromised by disruption of just one of these mechanisms.

### Whi3 Localizes to Structures Distinct from P-bodies and Stress Granules in Response to Heat Stress

Whi3 differed from a known stress granule component, Pub1, in the dynamics of its localization to stress granules following heat stress. Though stress granule assembly was required for Whi3 to form foci in response to glucose deprivation, Whi3 actually assembled into foci during mild heat stress, when Pub1 remained cytosolic. Whi3 colocalized with heat shock-responsive P-bodies during mild heat shock at 37°C and 39°C, suggesting that Whi3 may participate in P-body enhancement during mild heat shock. Furthermore, some Whi3 foci were distinct from foci containing the core P-body component Edc3 at 39°C, suggesting that Whi3 might localize to structures distinct from both P-bodies and stress granules during intermediate stress conditions. During heat shock at 42°C, when Pub1 began to appear in stress granules, Whi3 formed more foci and colocalized more strongly with the P-body marker Edc3 than with Pub1, suggesting that an increase in P-body size and number is a precursor to stress granule assembly in response to severe heat shock (Figures 3 and 4), as has been demonstrated during glucose deprivation [5].

The distinction between P-bodies and stress granules, particularly during stress, is still being elucidated. Ample evidence supports the model that both yeast and mammalian stress granules and P-bodies are related structures that share overlapping components [3], [5], [6], [21], [24], [26], [27], [37]. Consistent with this model, Whi3 exhibits behavior that incompletely overlaps with that of P-body or stress granule components. During steady-state conditions, Whi3 is not associated with foci, unlike P-body component Edc3. During mild heat stress, Whi3 localizes to puncta and partially colocalizes with Edc3, while stress granule component Pub1 remains diffuse. During extreme heat stress, Whi3 strongly colocalizes with Pub1. Further work is required to fully determine where Whi3 lies on the P-body/stress granule continuum.

Furthermore, stress granules show stress-specific compositions; though core stress granule components overlap, the assembly kinetics and composition of the granule vary in response to conditions such as glucose deprivation, heat shock, or sodium azide exposure [21], [26], [27], [37]. Our observation that Whi3 exhibits similar stress-responsive kinetics to Pub1 during glucose deprivation, but assembles in stress-responsive granules under heat stress conditions that do not induce Pub1 aggregation, agree with this model of stress-dependent variation in stress granule composition. During heat stress, Whi3 may be nucleating stress granules by participating in P-body enhancement at lower temperatures, and facilitating stress granule formation at higher temperatures.

Stress granules are stress-responsive hubs of RNA processing that sort non-translating transcripts and promote their decay, storage, or translational initiation [3], [6], [7], [27], [37]. The observation that Whi3 localizes to stress granules suggests that Whi3 mediates the fate of its target mRNAs during stress. The finding that Whi3-bound mRNAs are enriched for transcripts that function in cell cycle regulation and stress responses supports the current model of Whi3 participation in G_1_ regulation [9]–[11], [14], and suggests that Whi3 may promote survival in response to stress.

### A High-confidence Whi3 mRNA Target Set

Whi3 has a conserved RNA binding domain, but few Whi3 mRNA targets, with the exception of *CLN3*, have been examined in detail [8], [9]. Joint analysis of microarray data from our work and a preexisting data set produced a high-confidence list of Whi3 mRNA targets (Figure 5) [11]. GO term enrichment analyses of the high-confidence Whi3 target set suggested that Whi3 might be involved in several cellular processes aside from cell cycle regulation (Figure S1). Whi3 target mRNAs included transcription factors, regulators of rRNA transcription, and mediators of pheromone arrest. Our identification of a novel zinc sensitivity phenotype in *whi3*Δ cells suggests that zinc-related Whi3 mRNA targets may be misregulated in the absence of Whi3 (Figure 7). Examination of our high-confidence set of Whi3-interacting mRNAs suggested two candidates that could mediate this phenotype: *MSC2* and *ZAP1*. As Zap1 is a transcription factor involved in zinc homeostasis, it seemed the most likely Whi3-interacting mRNA to mediate the zinc-sensitivity phenotype. However, our observation that overexpression of Zap1 does not enhance the zinc sensitivity phenotype of *whi3*Δ cells (Figure 7) suggests that this phenotype may be due to misregulation of multiple Whi3 targets. As *whi3*Δ cells exhibit a variety of phenotypes indicative of misregulation of Whi3 target mRNAs (e.g., decreased mating efficiency and targets involved in pheromone response), the high-confidence list of Whi3 mRNA targets should be a useful starting point for future work characterizing non-cell cycle-related roles for Whi3.

### Whi3 Affects Target mRNA Abundance

We observed that levels of Whi3 target mRNAs were increased in *whi3*Δ cells relative to wild-type cells at both 30°C and 46°C. This observation suggests that Whi3 promotes target mRNA degradation, through a yet to be identified mechanism, that is apparently independent of its localization to stress granules.

The quantitative analysis of ribosomal association of mRNAs in the presence and absence of functional Whi3 presented here uncovered no evidence of translational regulation of its target mRNAs by Whi3, and is consistent with the hypothesis that Whi3 regulates expression of its mRNA targets primarily by promoting their degradation. However, because the translational data presented here were obtained from cells grown at steady-state conditions, it is possible that Whi3 regulates translation of its target mRNAs during stress.

### An Expanded Picture of Whi3-mediated Regulation

The majority of studies examining Whi3 function have focused on its role as a cell cycle regulator; the prevailing model of the primary function of Whi3 is that Whi3 mediates cell cycle entry by sequestering *CLN3* mRNA and Cdc28 protein in the cytosol [8], [10]–[12], [14]. Our observation that Whi3 localizes to stress granules in response to stress suggests a modified model: that Whi3 also regulates cell cycle entry by regulating the stability of its target mRNAs, and specifically *CLN3* mRNA through a mechanism which is yet to be determined. In support of this hypothesis, *whi3*Δ cells have significantly more *CLN3* mRNA than wild-type cells at 30°C (Figure 6B). As *CLN3* is the master regulator of the decision to pass Start, even small increases in *CLN3* gene dosage and/or expression result in advancement of Start, and a concomitant decrease in cell size [38], [39]. Thus, we predict that the increase in *CLN3* mRNA observed in *whi3*Δ cells results in elevated Cln3 protein level, and decreased cell size. Additional studies are required to determine if Whi3 causes *CLN3* mRNA to associate with stress granules, although the finding that Whi3 mediates aggregation of *CLN3* mRNA in *A. gossypii*, strongly suggests this possibility [36].

Expanding this model and the evidence for *CLN3* to some of the other mRNAs bound by Whi3, we speculate that Whi3 integrates environmental stimuli and coordinates the fates of several mRNAs that encode proteins required for the stress response. For example, Whi3-interacting mRNAs whose products function in glucose- or nutrient-regulated gene expression, such as *NRG1* or *PHO80*, may be regulated by Whi3 as part of the stress granule-mediated response to nutrient limitation. Whi3 may also mediate the response to other environmental changes such as pheromone arrest or metal ion toxicity through Whi3-interacting RNAs such as *POG1* or *AFT1*, respectively. *whi3*Δ is synthetically lethal with *ccw12*Δ, which lacks a critical cell wall component, and *whi3*Δ cells are sensitive to cell wall inhibitors; however Whi3 does not appear to be directly involved in cell wall assembly [11], [40]. The observation that Whi3 interacts with a variety of mRNAs involved in cell wall integrity [11], and our observation that Whi3 is a component of stress granules, suggests that Whi3 may mediate the response to cell wall stress. Future experiments should address the extent to which Whi3 is involved in these varied processes, and how much of Whi3-dependent RNA regulation is P-body and/or stress granule-dependent.

As this manuscript was being reviewed, other investigators similarly demonstrated that Whi3 has a small effect on the abundance of many of its mRNA targets, including *CLN3* [41]. Furthermore, results of these studies confirm that Whi3 localizes to stress granules and/or P-bodies in response to glucose deprivation and heat shock [41]. Taken together, these independent observations support our revised model of Whi3 function as a stress-dependent P-body/stress granule component that regulates abundance of many mRNA targets and promotes degradation of *CLN3* mRNA to regulate the cell cycle.

## Materials and Methods

### Yeast Strain Construction

All restriction digests and PCR for strain construction were performed using restriction enzymes and Vent polymerase from New England Biolabs. Whi3 was C-terminally tagged in the genome with 1xGFP-kanMX6 [42], mCherry-kanMX6 [43], or GST-kanMX6 [42] using previously described techniques [42].

To generate 3x-GFP integrated strains, the BamHI-3xGFP-NotI fragment from pB1963 [44] was inserted into integrating vector pRS303 [45] to create pRS303-3xGFP. Whi3 fragments incorporating base pairs 179 to 1983 (full length, FL), 179–738, 831–1983 (ΔQrich) or 179–1620 (ΔRRM) were amplified by PCR, digested with XhoI and BamHI-HF, and inserted into pRS303-3xGFP digested with XhoI and BamHI-HF. Whi3-3xGFP plasmids were linearized with EcoRV and transformed into yeast using standard protocols.

Whi3ΔQrich (lacking base pairs 739–830) was generated by overlap PCR. The N-terminal fragment (base pairs 174–738; 831–865) was amplified with KJ53 (5′CCGGctcgagCGTTGTGTCTAGTACCACCAACACAC3’; XhoI site in lower case) and KJ55 (5′GGCCTTGAGAAGATAACGGTATTGAAGAATTCACGCTTA AAGGAGAATCATTGGAAAATGG3’). The C-terminal fragment (base pairs 712–738; 831–1983) was amplified with KJ54 (5′CCATTTTCCAATGATTCTCCTTTAA GCGTGAATTC TTCAATACCGTTATCTTCTCAAGGCC3’) and KJ56 (5′CCGG ggatccCCGTTTTTTATATGACCAACATTAG3’; BamHI site in lower case). N- and C-terminal fragments were mixed in an equal molar ratio, then amplified using KJ53 and KJ56 to create Whi3ΔQrich; this cassette was digested with XhoI and BamHI-HF for insertion into pRS303-3xGFP. Whi3ΔRRM was amplified using KJ53 and KJ57 (5′-CCGGggatccCCAAGGGTATTACAAGGTGGATTTTGATCAGC-3′; BamHI site in lower case), digested with XhoI and BamHI, and inserted into pRS303-3xGFP.

### Fluorescence Microscopy

All microscopy experiments were performed using Openlab software 5.0.1 (Perkin Elmer-Cetus) and a Zeiss AxioImager M1 microscope (Carl Zeiss, Jena, Germany) coupled to a Hamamatsu Orca-ER digital camera (Bridgewater, NJ). Stress granule microscopy protocols were generally as described [26], [46], [47]. For all microscopy experiments, yeast were grown in 25 mL minimal media to OD600 of approximately 0.4. Cells were collected by centrifugation, washed in fresh media, and resuspended in fresh media pre-warmed to 30°C (glucose deprivation) or 46°C (heat shock). Cells were incubated in a water bath with shaking for 10–15 min, then 1 mL of culture was spun to pellet cells. Pelleted cells were resuspended in 50 µL of fresh, pre-warmed media, then 2.5 µL were spotted on a slide for immediate observation. To immobilize cells, coverslips were coated with Concanavalin A as previously described [46]. Coverslips were washed overnight in 1 M NaOH, then rinsed with sterile water until the pH was neutral. Coverslips were incubated in Concanavalin A solution (0.5 g/L Concanavalin A (Sigma), 10 mM phosphate buffer pH 6, 1 mM CaCl_2_, 0.02% sodium azide) for 20 min at room temperature with gentle shaking. Coverslips were rinsed once in sterile water and air-dried vertically.

### Stress Granule Pelleting Assay

The pelleting assay protocol was adapted from [4], [26]. Exponentially growing cultures were split into two and resuspended in fresh media pre-warmed to 30°C (control) or 46°C (heat shock). Cells were incubated for 10 min at the appropriate temperature, then harvested by centrifugation and washed in 1 mL of sterile water. Cell pellets were resuspended in lysis buffer (50 mM Tris HCl pH 7.6, 50 mM NaCl, 5 mM MgCl_2_, 0.1% NP-40, 1 mM ß-mercaptoethanol, 5 mM DTT, 1 mM PMSF, 10 µg/mL leupepstatin, 10 µg/mL aprotinin, 6.25 mM benzamidine, 2.5 µg/mL pepstatin). Glass beads were added to the meniscus, and cells were vortexed 6×1 min with 1 min rests at 4°C. Extract was clarified by centrifugation at 2000×g, 2 min, 4°C; extract was transferred to a fresh eppendorf tube and cellular debris was discarded. Extract was centrifuged at 10,000×g, 10 min, 4°C. Supernatant was removed from the pellet, which was resuspended in lysis buffer. Supernatant and pellet fractions were analyzed by Western blotting.

### Isolation and Analysis of Whi3 mRNA Targets

Whi3 was affinity purified and associated RNAs were identified by microarray analysis, essentially as previously described [29]. TAP-tagged yeast strains derived from BY4741 (Open Biosystems Cat# YSC1177) were grown to an OD600 of 0.6–0.8 in minimal media (6.7 g Difco Yeast Nitrogen Base without amino acids, 60 mg L-leucine, 20 mg L-histidine, 20 mg L-methionine, 20 mg uracil, and 20 g glucose per liter) or YPD. For each IP, cells growing at mid-log phase were harvested by centrifugation, washed twice with Buffer A (50 mM HEPES pH 8.0, 140 mM KCl, 1.8 mM MgCl_2_, 0.1% NP-40, and 0.2 mg/mL heparin), resuspended in Buffer B (Buffer A with 1 µg/mL pepstatin, leupeptin, and vanadate, 2.5 µg/mL aprotinin, 1 mM PMSF, 0.5 mM DTT, and 0.1U/uL Superasin RNase inhibitor from Ambion), and lysed at 4°C by mini bead-beater 8 from Biospec products (Cat# 693) with four 1 min cycles at max speed. Lysate was cleared by centrifugation for 10 min at 8,000×g and 4°C, and total protein concentration was adjusted to 15 mg/mL by dilution with Buffer B. Biotinylated rabbit IgG was coupled to streptavidin coated magnetic beads (Invitrogen Cat# 602-10). Beads were incubated with lysate for 2 hours, then washed for 15 min on a rotator at 4°C, once with buffer B and three times with Buffer C (Buffer B with 10% glycerol and no heparin or vanadate). 100 µL of the lysate remaining after the beads were removed was set aside for the isolation of reference RNA. IP RNA was isolated with phenol:chloroform as described [48]. Total RNA for use as a reference was purified from the lysate remaining after the 2 hour incubation with the beads, using PureLink Micro-to-Midi Kit (Invitrogen Cat# 12183-018).

Whi3 was purified as described above, and one-class SAM analysis was used to identify targets [29]. To define a high-confidence Whi3 target set, we selected mRNAs that were called Whi3 targets from both datasets at a false discovery rate <1% and that also had the previously identified Whi3 sequence motif.

Whi3 IP data from Colomina et al. 2008 were re-analyzed using the Significance Analysis of Microarrays (SAM) algorithm to compare the Whi3 IP to a mock IP using a two-class analysis [29], [30].

GO term enrichment was calculated using the GO stats package in R, and the resulting p-values were corrected for multiple hypothesis testing with the Bonferroni correction [31].

### DNA Microarray Production and Pre-hybridization Processing

Yeast DNA microarrays were printed on epoxysilane-coated glass (Schott Nexterion E) by the Stanford Functional Genomic Facility. Further information about the probes used, including probe sequences, is available from the Operon Web site (https://www.operon.com/; *S. cerevisiae* YBOX V1.0).

Detailed protocols for microarray experiments can be found on the Brown Lab website (http://cmgm.stanford.edu/pbrown/protocols/index.html). The microarray prehybridization performed has been previously described. Within 24 hours prior to hybridization, slides were placed in a humidity chamber (Sigma Cat# H6644) filled with 100 mL of 0.56x SSC (16x SSC = 150 mM NaCl, 15 mM sodium citrate [pH 7.0]) for 30 min at room temperature. Slides were then dried rapidly at 70–80°C on a heat block. The epoxysilane surface of the slides was blocked by incubation with 1 M Tris-HCl (pH 9.0), 100 mM ethanolamine, and 0.1% SDS for 20 min at 50°C. After blocking, the slides were washed twice for 1 min with 400 ml of water, and then dried by centrifugation.

### DNA Microarray Sample Preparation, Hybridization, and Washing

Reference RNA from extract and affinity-purified RNA from the Whi3 IP were reverse transcribed with Superscript II (Invitrogen Cat# 18064–014) in the presence of 5-(3-aminoallyl)-dUTP (Ambion Cat# AM8439) and natural dNTPs (GE Healthcare Life Sciences Cat# US77212) with a 1∶1 mixture of N9 and dT20V primers (Invitrogen). Subsequently, amino-allyl–containing cDNAs were covalently linked to Cy3 and Cy5 NHS-monoesters (GE Healthcare Life Sciences Cat# RPN5661). Dye-labeled DNA was diluted in a 20–40-ll solution containing 33x SSC, 25 mM HEPES-NaOH pH 7.0, 20 µg of poly(A) RNA (Sigma cat # P4303), and 0.3% SDS. The sample was incubated at 95.8°C for 2 min, spun at 14,000rpm for 10 min in a microcentrifuge, and then hybridized at 65°C using the MAUI hybridization system (BioMicro) for 12–16 h. After hybridization, slides were washed first in a solution of 2x SSC with 0.05% SDS at 70°C for 5 min, then in 2x SSC at room temperature for 2 min, then in 1x SSC at room temperature for 2 min, then in 0.2x SSC at room temperature for 2 min. Slides were then dried by centrifugation in a low- ozone room (<5ppb).

### DNA Microarray Scanning and Data Processing

Microarrays were scanned using an AxonScanner 4000B (Molecular Devices). PMTs were adjusted to maximize signal, without excessive background and pixel saturation. Microarray spots were located and their data extracted using the GenePix Pro 6.0 software (Molecular Devices). All data is MIAME compliant and the raw data has been deposited in a MIAME compliant database. The data were filtered for signal vs. background using several parameters. Specifically, the Cy5 (red) vs Cy3 (green) pixel intensity values for each spot must have a correlation coefficient (R-squared) 0.6. In addition, the signal intensity minus the local background for each spot must be greater than 100, or greater than 3x the standard deviation of the local background surrounding each spot. Signal in either channel that failed these filtering criteria was considered absent. Spots with green signal but no red signal were kept separated as RNAs that were expressed but did not co-purify with Whi3. Finally, both the technical replicates of each DNA oligonucleotide (each oligonucleotide was printed twice per microarray) had to pass filtering for that spot to be considered as a possible target of Whi3. The log (base 2) of the Cy5 to Cy3 ratio (Log2 Ratio or L2R) for each spot that passed filtering was used for the subsequent analyses.

### Translational Analysis of Whi3 Targets

Translation analysis was performed essentially as described previously [32], except for the following details. Three individual cultures of wild-type or *whi3*Δ cells were grown to OD600 0.6 in 500 mL YPD. Cultures were divided into 2×250 mL cultures, and incubated at 30°C or 46°C for 15 min. 1/100 volume of 10 mg/mL cycloheximide was added, and cultures were shaken for 30 sec before cells were harvested and washed in ice-cold buffer (50 mM HEPES pH 8.0, 140 mM KCl, 5 mM MgCl_2_, 0.1% NP-40, 0.02 mg/mL heparin, and 0.1 mg/mL cycloheximide). Pelleted cells were resuspended in 500 µL buffer and transferred to 1.5 mL screw-cap tubes with 700 µL glass beads. Cells were lysed in a bead beater in 4×1 min cycles with 1 min rests on ice. Lysates were clarified by centrifugation. Equal volumes of cell lysate containing 400 µg RNA were loaded onto a 5–50% w/vol sucrose gradient (prepared as described, [32]) and centrifuged at 41,000 RPM for 1.5 h at 4°C. From this point, the experiment proceeded exactly as described in [32].

### RT-PCR

Cells were grown as for microscopy experiments to an OD600 of 0.3 in 50 mL YPD media. Five individual cultures per cell type (wild-type or *whi3*Δ) were used for RT-PCR analysis. Cells were heat-shocked as described above, and total mRNA was isolated by hot phenol extraction [48]. cDNA was amplified using random primers (Invitrogen, Cat. No. 48190-011). All cDNA was diluted 1∶25, and thereafter into a dilution series of four 1∶5 dilutions. All samples were analyzed in two technical replicates for each gene. RT-PCR reactions consisted of 2 µL cDNA, 3 µL 1.5 µM primers, and 5 µL Power SYBR**®** Green PCR master mix (Applied Biosystems, Cat. No. 4368708). Samples were run on a 7900 HT Fast Real-Time PCR System (Applied Biosystems) using SDS 2.3 software. Data analysis was performed in Microsoft Excel for Mac 2011. Dilution series for each gene were plotted on a log scale to determine a critical threshold (Ct) value for each sample. Values from technical replicates were averaged. All samples were first normalized to their own ACT1 levels, then normalized to the wild-type 30°C condition. Error bars represent SEM from three biological replicates.

### Zinc Sensitivity Growth Assays

Yeast cells were grown to OD600≥2.0 in SCD media (untransformed cells) or SCD lacking uracil (transformed cells). Cells were washed thoroughly in water then diluted to equal density in 260 µL sterile water. A dilution series of six 1∶5 dilutions was spotted onto petri plates containing SCD media containing 0, 10 mM, or 15 mM zinc. Once plates were dry, they were incubated at 30°C for 2–5 days until growth phenotypes could be observed.

## Supporting Information

Figure S1 **GO term analysis of high-confidence Whi3-interacting RNAs.** Heat map of the GO term enrichment for sets of Whi3-interacting RNAs. The green squares indicate a significant enrichment of a GO term for a particular target set (after Bonferroni correction for multiple hypothesis testing). Darker green corresponds to a more significant enrichment.
(EPS)

Table S1 **This table shows the mRNAs that were called targets by analysis of our immunoprecipiation data, our analysis of the data published by Colomina et al** [**11**] **, or both.** The False discovery rate (FDR) reported by the SAM algorithm was used to call mRNA targets from each data set. We used an FDR cutoff of 1%, corresponding to a 1% chance that a target identified at this threshold is a false discovery. The score given by the SAM algorithm indicates the degree of mRNA enrichment in the immunoprecipitated sample and takes into account the reproducibility. The top 100 high-confidence mRNA targets that had an FDR <1% in both data sets and also have the Whi3 sequence motif are shown with their relative rank.
(XLS)
